# The genomic analysis brings a new piece to the molecular jigsaw of idiopathic erythrocytosis

**DOI:** 10.1186/s40164-022-00301-1

**Published:** 2022-08-28

**Authors:** Antonella Zagaria, Francesco Tarantini, Paola Orsini, Luisa Anelli, Cosimo Cumbo, Nicoletta Coccaro, Giuseppina Tota, Crescenzio Francesco Minervini, Elisa Parciante, Maria Rosa Conserva, Immacolata Redavid, Alessandra Ricco, Immacolata Attolico, Giorgina Specchia, Pellegrino Musto, Francesco Albano

**Affiliations:** 1 Department of Emergency and Organ Transplantation (D.E.T.O.), Hematology and Stem Cell Transplantation Unit, University of Bari “Aldo Moro”, P.Zza G. Cesare, 11, 70124 Bari, Italy; 2 School of Medicine, University of Bari “Aldo Moro”, 70124 Bari, Italy

**Keywords:** Erythrocytosis, Myeloproliferative neoplasms, SNPs, JAK2, EPO

## Abstract

Erythrocytosis is a clinical condition characterized by increased red cell mass, hemoglobin, and hematocrit values. A significant fraction of patients is described as having idiopathic erythrocytosis. We have previously demonstrated an association between erythrocytosis and the *JAK2* GGCC_46/1 haplotype and *CALR* rs1049481_G allele. In the present study, we investigated genomic and clinical features of 80 erythrocytosis patients with the aim to provide useful information in clinical practice. Patients with idiopathic erythrocytosis could have a genomic germline background, eventually associated with somatic variants. Through association analysis, we show that male patients presenting with idiopathic erythrocytosis, and normal EPO levels could be the best candidates for the search for the *JAK2* GGCC_46/1 haplotype and *CALR* rs1049481_G allele. Further studies are needed to confirm these findings and to depict detailed genomic and phenotypical characteristics of these patients.

**Supplementary Information:**

The online version contains supplementary material available at 10.1186/s40164-022-00301-1.

To the editor,

Erythrocytosis is characterized by an erythrocyte count above the gender specific normal range and increased hemoglobin and hematocrit values [1]. Polycythemia vera (PV) accounts for most primary acquired erythrocytosis cases; the *JAK2* V617F or *JAK2* exon 12 variants are considered PV “driver” mutations. However, about 4% of PV cases lack a molecular marker [1, 2].

Although recent evidence has added useful information to define erythrocytosis [3, 4] a significant fraction of patients is described as affected by idiopathic erythrocytosis (IE), characterized by a genetic marker absence; the IE clinical management still represents an unmet need. We previously demonstrated an association between erythrocytosis and two single nucleotide polymorphisms (SNPs): *JAK2* GGCC_46/1 and *CALR* rs1049481_G [5]. In this study, we investigated genomic and clinical features of a larger cohort of patients to unveil the IE molecular complexity (Additional file 1). Based on clinical and genomic data of a more extensive patient’s cohort, we suggest a hierarchical model in which male patients presenting with IE and normal erythropoietin (EPO) levels are the best candidates for the search for *JAK2* and *CALR* SNPs. Furthermore, in this subset of patients, we identified additional mutations in genes commonly involved in clonal hematopoiesis (CH).

The *JAK2* and *CALR* SNPs were genotyped in 80 cases (Additional file 2: Table S1) as previously described [5]. Fifty-three (66.3%) were positive and 27 (33.7%) negative for the *JAK2* haplotype. Regarding *CALR*, 54 (67.5%) cases had at least one G allele.

The *JAK2* SNP was associated with erythrocytosis, a significant difference in frequency being detected as compared to healthy European controls (p = 0.0011). The association was also demonstrated in terms of allelic frequency (p = 0.0019) and genotype distribution (p = 0.0035).

The simultaneous presence of both SNPs was observed in 38 (47.5%) cases compared to controls (137/503, 27.2%) (p = 0.0004). A significant association between SNPs and erythrocytosis was also observed in cases showing normal EPO (p = 0.0002).

Since both SNPs are in accordance with Hardy–Weinberg equilibrium in controls (*p* > 0.05), association analysis was performed between the SNPs investigated and erythrocytosis using the SNPassoc R package [6]. A significant association between *JAK2* SNP and erythrocytosis risk was observed under the dominant model, with a 2.29-fold higher risk in people bearing at least one alternative allele compared to subjects having none (OR = 2.29; *p* = 0.0007576) (Table 1). Considering *CALR*, the presence of at least one G allele is associated with an increased risk under a log-additive model (0,1,2 G: OR = 1.37; *p* = 0.06609).

**Table 1:** Associations between *JAK2* GGCC_46/1 haplotype and erythrocytosis cases

SNP	Genotype	Control (503)	Case (80)
rs3780367	HWE = 0.6868	HWE = 0.4974	HWE = 0.3501
	T/T	271 (53.9%)	27 (33.8%)
	T/G	192 (38.2%)	43 (53.8%)
	G/G	40 (8%)	10 (12.5%)

Genetic inherytance model	OR (95% CI)	p-value	AIC
Codominant	2.23 (1.33, 3.74)	0.0036866	461.1
Dominant	2.29 (1.4, 3.76)	0.0007576	458.9
Recessive	1.65 (0.79, 3.46)	0.1991234	468.6
Overdominant	1.88 (1.17, 3.03)	0.0089603	463.4
log-Additive	1.75 (1.23, 2.47)	0.0019041	460.6
Adjustment by single covariates			
CALR rs1049481_G (yes/no)	2.3 (1.4, 3.78)	0.0007354	459.5
Gender	2.62 (1.54, 4.43)	0.000255	362.9
Epo level	2.73 (1.59, 4.68)	0.000153	417.1
Adjustment by multiple covariates			
Sex-Epo level and *CALR* rs1049481_G (yes/no)	3.13 (1.76, 5.5)	0.000051	331.9

To improve the accuracy of the test, several covariates were incorporated; the association became stronger after adjustment for the presence of *CALR* rs1049481_G as a categorical variable, as well as gender, and EPO level (Table 1). The erythrocytosis risk is higher when the three covariates are introduced simultaneously (OR = 3.13, p = 0.000051; Table 1). Considering patients with normal EPO levels, all observed associations between *JAK2* SNP and erythrocytosis under the dominant model were strengthened (with *CALR* rs1049481_G as covariate: OR = 2.75, p = 0.0001381; with gender: OR = 3.11, p = 0.0000522).

Next generation sequencing (NGS) analysis was performed on 44 patients; 34/44 (77%) sequenced cases with the *JAK2* haplotype showed at least one allele G of *CALR* rs1049481. Overall, 22 genetic variants affecting 7 genes (*ASXL1*, *TET2*, *DNMT3A*, *JAK2*, *KIT*, *RUNX1*, ANKRD26) were detected in 17/44 cases (38.6%) (Fig. 1A). *ASXL1* was the most frequently mutated gene (6/44, 14%) (Fig. 1A, B). Two non-canonical *JAK2* variants were identified (Additional file 3: Table S2), already described in few patients with haematologic neoplasms [7].

**Fig. 1:**
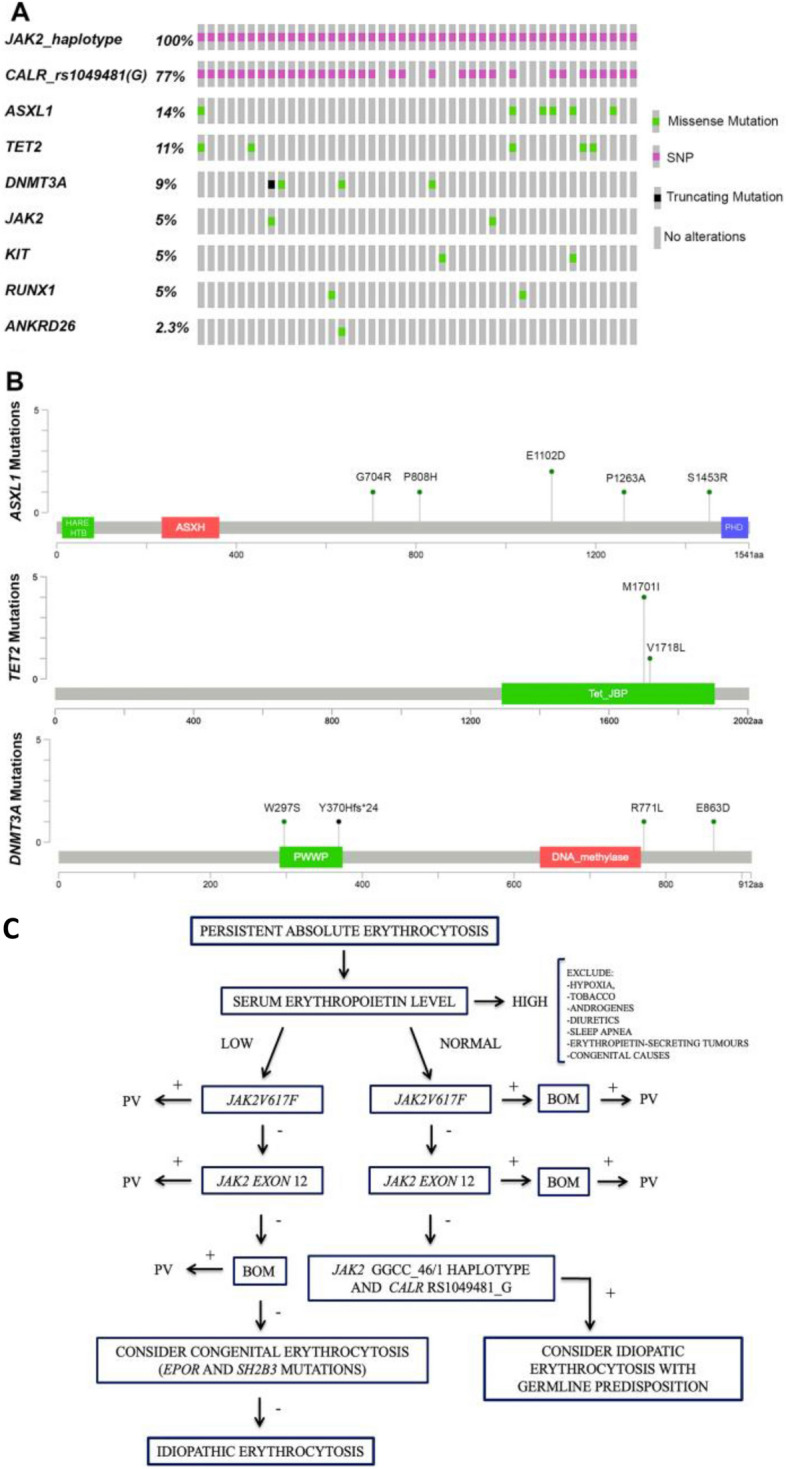
**A** Oncoprint visualization of all genetic variants identified by targeted NGS analysis in 44 erythrocytosis cases. SNP: single nucleotide polymorphism. **B** Maps of the mutations on linear proteins of the most mutated genes in all sequenced cases. Green dots stand for missense mutations, while black dots indicate frameshift mutations. The height of the bar depends on the number of cases bearing each variant. HARE-HTH: HB1, ASXL, restriction endonuclease HTH domain (12–83); ASXH: Asx homology domain (234–362); PHD: PHD domain of transcriptional enhancer, Asx (1480–1539); PWWP: Pro-Trp-Trp-Pro domain (291–374); DNA_methylase: C-5 cytosine-specific DNA methylase (634–767); Tet_JBP: Oxygenase domain of the 2OGFeDO superfamily (1290–1905). **C** Diagnostic approach to erythrocytosis patients. PV: polycythemia vera, BOM: bone marrow biopsy

Recent evidence suggests that germline predisposition factors could have a role in the development of myeloproliferative neoplasms [3, 8–10]. Based on the integration of genomic data, clinical features, and statistical methodology, we have attempted to refine the typical characteristics of patients presenting with IE. The median age of our patients with typical CH genes mutations was 52 years (only 2 patients were > 60 years). Therefore, such mutations cannot be attributed to an aging-related CH [11].

We hypothesize that a degree of genomic instability could create a “fertile ground” for the development of erythrocytosis, characterized by a high prevalence of additional mutations in typical CH genes*.* Furthermore, association analysis builds a sort of genomic hierarchy, prioritizing the presence of *JAK2* GGCC_46/1 over the *CALR* rs1049481_G allele. Finally, male patients with IE and normal EPO levels are more likely to benefit from the analysis of both *JAK2* and *CALR* SNPs to better define the challenging diagnostic process of IE (Fig. 1C). Further studies are needed to confirm these findings and to depict detailed characteristics of IE patients.

## Supplementary Information

**Additional file 1.** Methods.

**Additional file 2: Table S1.** Biological and clinical characteristics of cases analyzed in the present study.

**Additional file 3: Table S2.** Variants identified by NGS analysis in 44 erythrocytosis cases.

## Data Availability

The sequence data from this study have been submitted to the National Center for Biotechnology Information (NCBI) Short Read Archive (https://www.ncbi.nlm.nih.gov/sra/) under accession number PRJNA609847.

## Abbreviations

PV: Polycythemia vera
IE: Idiopathic erythrocytosis
SNP: Single nucleotide polymorphism
EPO: Erythropoietin
CH: Clonal hematopoiesis
NGS: Next generation sequencing

## References

[1] Arber DA, Orazi A, Hasserjian R, Thiele J, Borowitz MJ, le Beau MM (2016). The 2016 revision to the World Health Organization classification of myeloid neoplasms and acute leukemia. Blood.

[2] Storlazzi CT, Albano F, Locunsolo C, Lonoce A, Funes S, Guastadisegni MC (2006). t(3;12)(q26;q14) in polycythemia vera is associated with upregulation of the HMGA2 gene. Leukemia.

[3] Gašperšič J, Kristan A, Kunej T, Zupan IP, Debeljak N (2021). Erythrocytosis: genes and pathways involved in disease development. Blood Transfus.

[4] Olcaydu D, Harutyunyan A, Jäger R, Berg T, Gisslinger B, Pabinger I (2009). A common JAK2 haplotype confers susceptibility to myeloproliferative neoplasms. Nat Genet.

[5] Anelli L, Orsini P, Zagaria A, Minervini A, Coccaro N, Parciante E (2020). Erythrocytosis with JAK2 GGCC_46/1 haplotype and without JAK2 V617F mutation is associated with CALR rs1049481_G allele. Leukemia.

[6] González JR, Armengol L, Solé X, Guinó E, Mercader JM, Estivill X (2007). SNPassoc: an R package to perform whole genome association studies. Bioinformatics.

[7] Cumbo C, Tarantini F, Zagaria A, Anelli L, Minervini CF, Coccaro N (2022). Clonal hematopoiesis at the crossroads of inflammatory bowel diseases and hematological malignancies: a biological link?. Front Oncol.

[8] Bento C (2018). Genetic basis of congenital erythrocytosis. Int J Lab Hematol.

[9] Camps C, Petousi N, Bento C, Cario H, Copley RR, McMullin MF (2016). Gene panel sequencing improves the diagnostic work-up of patients with idiopathic erythrocytosis and identifies new mutations. Haematologica.

[10] Wouters HJCM, Mulder R, van Zeventer IA, Schuringa JJ, van der Klauw MM, van der Harst P (2020). Erythrocytosis in the general population: clinical characteristics and association with clonal hematopoiesis. Blood Adv.

[11] Jaiswal S, Fontanillas P, Flannick J, Manning A, Grauman PV, Mar BG (2014). Age-related clonal hematopoiesis associated with adverse outcomes ABSTRACT. N Engl J Med.

