# Supplementary material for: The genomic analysis brings a new piece to the molecular jigsaw of idiopathic erythrocytosis
Source: Exp Hematol Oncol. 2022 Aug 28;11:47. doi: 10.1186/s40164-022-00301-1 (PMC9420251; doi:10.1186/s40164-022-00301-1)
Supplement: Supplementary file 1 — Additional file 1. Methods. [file 40164_2022_301_MOESM1_ESM.docx]

**Methods**

*Patients and Controls*

The study included 80 patients diagnosed with erythrocytosis from 2009 to 2022. Patients were selected based on WHO-suggested Hb (>16.5 g/dL in men, >16.0 g/dL in women) and HCT (>49%in men, >48% in women) cut-offs. All patients were negative for canonical *JAK2* mutations and secondary causes. All cases were checked for germline mutations in genes known to cause erythrocytosis and the *BCR-ABL1* rearrangement [1]. Most patients (78/80, 97.5%) were male, with a median age of 50 years (range 19-72). Based on the erythropoietin level, erythrocytosis patients were subdivided into normal (70 cases) and subnormal level (10 cases). These latter were subsequently diagnosed with Polycythemia Vera according to World Health Organization 2016 criteria.

*In silico* data from 503 healthy European subjects of the 1000 Genomes (1000G) Project were used as control group, and the occurrence of the *JAK2* GGCC_46/1 haplotype was determined using the tagging SNP rs3780367.

The study was approved by the local ethics committee “Azienda Ospedaliero Universitaria Policlinico di Bari”. Written informed consent was obtained from all patients before enrollment in accordance with the Declaration of Helsinki.

*Genotyping Methods*

Genomic DNA from all 80 patients was extracted from peripheral blood (PB) granulocytes. The *JAK2* V617F mutation and rare mutations in *JAK2* exon 12 were shown to be absent using a real-time quantitative PCR assay and amplification and subsequent Sanger sequencing of the entire exon 12. The presence of the *JAK2* GGCC_46/1 haplotype and *CALR* rs1049481_G was investigated by PCR analysis [2].

*Statistical Analysis*

Fisher’s exact test was used to compare the frequency distribution of the *JAK2* GGCC_46/1 haplotype and *CALR* rs1049481 in erythrocytosis patients and controls, respectively. Calculation of the Hardy-Weinberg equilibrium (HWE) and analysis of association between SNPs and erythrocytosis based on generalized linear models was performed with the SNPassoc R package (https://cran.r-project.org/web/packages/SNPassoc/index.html) [3]. The HWE for both polymorphisms was assessed in controls. The association was measured by the odds ratio and 95% confidence interval (considering the most frequent homozygous genotype as the reference), P-value and the Akaike Information Criterion (AIC) under five genetic models (codominant, dominant, recessive, overdominant and log-additive). The best genetic model was obtained via AIC. Statistical significance was set at p<0.05. Some covariates were incorporated into association tests. The EPO level was assumed to be normal in the control group (503 individuals).

*Next-generation sequencing analysis*

Forty-four among 80 (55%) erythrocytosis cases were selected for further next generation sequencing (NGS) analysis as they were still in follow-up at our Hematology Unit. Their median age was 52 years (range 21-72). NGS analysis with an AmpliSeq customized panel (Thermo Fisher Scientific), encompassing 26 target genes that are frequently mutated in myeloid malignancies, was performed on genomic DNA extracted from PB samples [4]. Quality control reads alignment to the human genome (hg19), and variant calling (using the somatic workflow for single samples and the default parameters) were performed using Torrent Suite Software v5.16 (Thermo Fisher Scientific). Variants were annotated using Ion Reporter Software v5.16 (Thermo Fisher Scientific). Variants located in intronic regions (not in splice sites) or synonymous, or present with >1% global minor allele frequency in the healthy population, according to the 1000G database, were filtered out. The resulting single nucleotide variants and insertions/deletions were then filtered out if detected in a control sample (Human CEPH Genomic DNA Control by Thermo Fisher; NC). Only variants with ≥2% variant allele frequency and with a depth of coverage >500x were considered. For all detected variants, no artefacts were observed at visual inspection of .bam files by the Integrative Genomics Viewer tool [5].

**References**

1. Bento C. Genetic basis of congenital erythrocytosis. International Journal of Laboratory Hematology [Internet]. John Wiley & Sons, Ltd; 2018 [cited 2022 May 11];40:62–7. Available from: https://onlinelibrary.wiley.com/doi/full/10.1111/ijlh.12828

2. Anelli L, Zagaria A, Specchia G, Albano F. The JAK2 GGCC (46/1) Haplotype in Myeloproliferative Neoplasms: Causal or Random? International Journal of Molecular Sciences 2018, Vol 19, Page 1152 [Internet]. Multidisciplinary Digital Publishing Institute; 2018 [cited 2022 May 11];19:1152. Available from: https://www.mdpi.com/1422-0067/19/4/1152/htm

3. González JR, Armengol L, Solé X, Guinó E, Mercader JM, Estivill X, et al. SNPassoc: an R package to perform whole genome association studies. Bioinformatics [Internet]. Bioinformatics; 2007 [cited 2022 May 11];23:644–5. Available from: https://pubmed.ncbi.nlm.nih.gov/17267436/

4. Cumbo C, Tota G, de Grassi A, Anelli L, Zagaria A, Coccaro N, et al. RUNX1 gene alterations characterized by allelic preference in adult acute myeloid leukemia. Leukemia and Lymphoma [Internet]. Taylor and Francis Ltd.; 2021 [cited 2022 Jan 26]; Available from: https://www.researchgate.net/publication/351813029_RUNX1_gene_alterations_characterized_by_allelic_preference_in_adult_acute_myeloid_leukemia

5. Robinson JT, Thorvaldsdóttir H, Winckler W, Guttman M, Lander ES, Getz G, et al. Integrative Genomics Viewer. Available from: www.1000genomes.org/
